# Associations of self-reported and actigraphic sleep with gut microbiome composition and diversity among older adults

**DOI:** 10.1093/sleep/zsag217

**Published:** 2026-08-08

**Authors:** Curtis Tilves, Calliope Holingue, Sarah K Wanigatunga, Chee W Chia, Ni Zhao, Mark N Wu, Jennifer A Schrack, Eleanor M Simonsick, Luigi Ferrucci, Toshiko Tanaka, Adam P Spira, Noel T Mueller

**Affiliations:** Department of Epidemiology, Colorado School of Public Health, University of Colorado Anschutz Medical Campus, Aurora, CO, United States; Lifecourse Epidemiology of Adiposity and Diabetes (LEAD) Center, University of Colorado Anschutz Medical Campus, Aurora, CO, United States; Department of Mental Health, Johns Hopkins Bloomberg School of Public Health, Baltimore, MD, United States; Center for Autism Services, Science and Innovation, Kennedy Krieger Institute, Baltimore, MD, United States; Department of Mental Health, Johns Hopkins Bloomberg School of Public Health, Baltimore, MD, United States; Intramural Research Program, National Institute on Aging, National Institutes of Health (NIH), Bethesda, MD, United States; Department of Biostatistics, Johns Hopkins Bloomberg School of Public Health, Baltimore, MD, United States; Department of Neurology, Johns Hopkins School of Medicine, Baltimore, MD, United States; Department of Epidemiology, Johns Hopkins Bloomberg School of Public Health, Baltimore, MD, United States; Center on Aging and Health, Johns Hopkins University, Baltimore, MD, United States; Intramural Research Program, National Institute on Aging, National Institutes of Health (NIH), Bethesda, MD, United States; Intramural Research Program, National Institute on Aging, National Institutes of Health (NIH), Bethesda, MD, United States; Intramural Research Program, National Institute on Aging, National Institutes of Health (NIH), Bethesda, MD, United States; Department of Mental Health, Johns Hopkins Bloomberg School of Public Health, Baltimore, MD, United States; Center on Aging and Health, Johns Hopkins University, Baltimore, MD, United States; Department of Psychiatry and Behavioral Sciences, Johns Hopkins School of Medicine, Baltimore, MD, United States; Department of Pediatrics-Nutrition, University of Colorado School of Medicine Anschutz Medical Campus, Aurora, CO, United States

**Keywords:** sleep, insomnia, aging, microbiome, actigraphy

## Abstract

**Study Objectives:**

Poor sleep is linked to adverse health outcomes. Animal studies suggest the gut microbiome may influence sleep, but human findings remain inconsistent. We examined associations of self-reported insomnia symptoms, daytime sleepiness, and actigraphy-measured sleep with gut microbiome diversity and composition in older adults.

**Materials and Methods:**

We studied 869 Baltimore Longitudinal Study of Aging participants with self-reported sleep and shotgun metagenomic sequencing; 332 also had actigraphy. We tested associations of sleep with alpha diversity, beta diversity, and species composition using regression, PERMANOVA, and ANCOM-BC2, adjusting for age, sex, BMI, physical activity, education, and depressive symptoms.

**Results:**

Participants had a mean age of 70.7 years; 54.8 per cent were female and 66.9 per cent white. Trouble falling asleep ≥5 times/week was associated with higher Shannon diversity (*β* = 0.41 SD; 95% CI: 0.09, 0.73) and Pielou’s evenness, but not richness metrics. No actigraphy-measured sleep variables were associated with alpha or beta diversity. Beta diversity analyses suggested excessive sleepiness (1–2 or 3–4 times/week) was associated with a different microbial composition, though the variance explained was small. In species-level analyses, frequent insomnia symptoms or excessive sleepiness were associated with depleted or undetected *Eubacterium* sp. CAG:251. In exploratory actigraphy models, each doubling of sleep efficiency was associated with higher *Eubacterium* sp. CAG:251 prevalence (PR = 2.15; 95% CI: 1.47, 3.14), while each 30-minute increase in wake after sleep onset was associated with lower prevalence (PR = 0.49; 95% CI: 0.29, 0.81).

**Conclusions:**

Global diversity findings were limited and inconsistent, whereas subjective and objective sleep disturbances converged on *Eubacterium* sp. CAG:251. These findings are exploratory and require longitudinal replication.

## Teaser

Sleep disturbances are highly prevalent in older adults and are linked to adverse physical and cognitive outcomes, yet the biological pathways connecting sleep and health remain poorly understood. The gut microbiome may contribute to sleep regulation, but prior human studies have generally been small, used limited sleep measures, and relied on 16S rRNA sequencing. In a larger, well-characterized cohort of older adults, we combined self-reported insomnia symptoms, actigraphy-derived sleep metrics, and shotgun metagenomic sequencing to examine gut microbial diversity and species-level composition. Global diversity findings were limited and inconsistent, but subjective and objective sleep disturbances converged on *Eubacterium* sp. CAG:251. These hypothesis-generating findings identify a candidate species-level taxon for replication and future longitudinal and mechanistic studies.

## Graphical Abstract

**Graphical Abstract:**
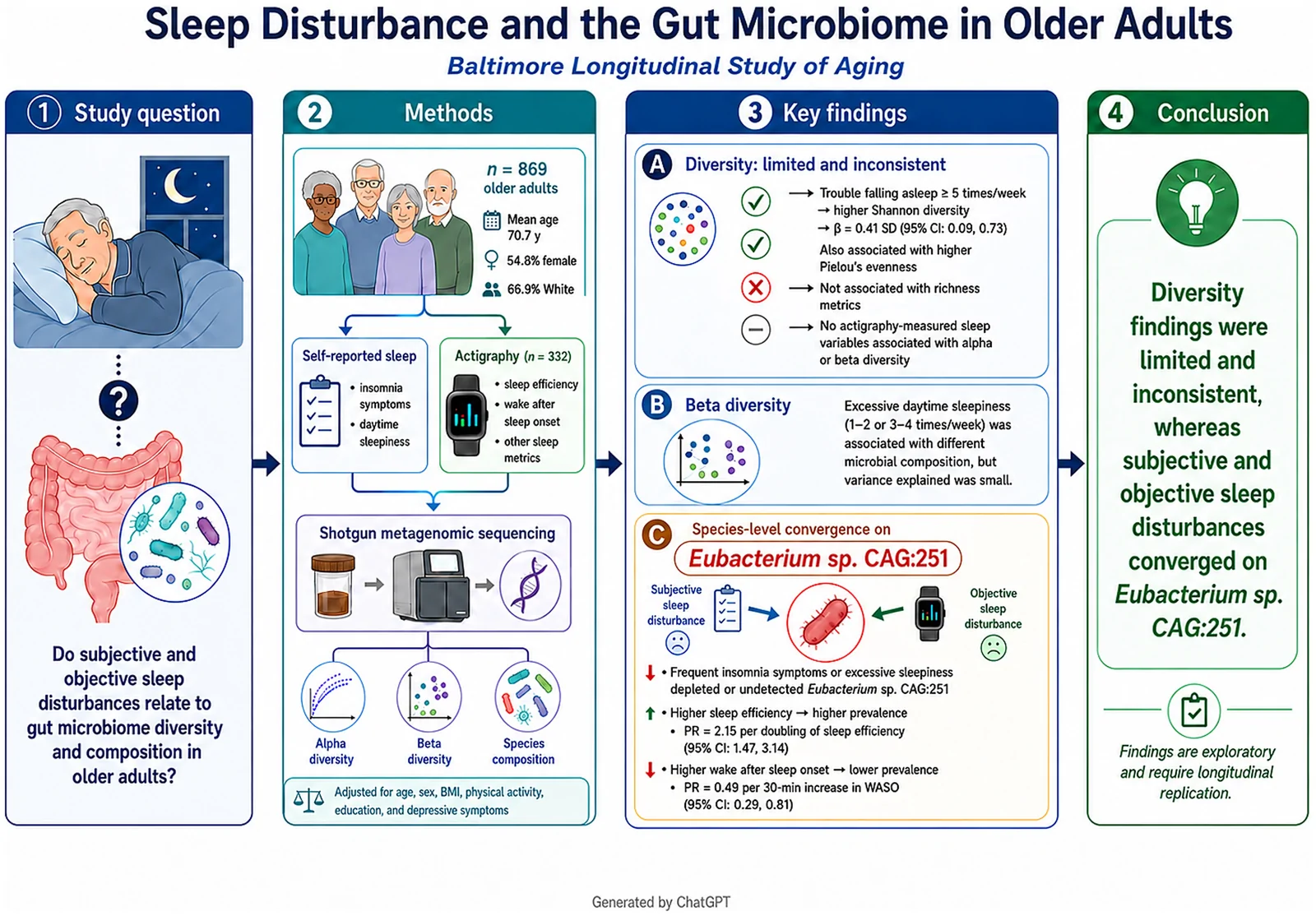

## Introduction

Sleep disturbances in later life encompass a range of issues, including insomnia symptoms (subjective dissatisfaction with sleep quality, difficulty falling or staying asleep, waking too early in the morning, or non-restorative sleep) as well as objectively measured changes such as greater sleep fragmentation and lower sleep efficiency (the proportion of time in bed spent asleep). These disturbances become more common with advancing age, with about 20%–40% of older adults reporting sleep difficulties at least a few nights a month [1–4]. A study of 9000 adults aged 65 and older found that only 12 per cent reported no sleep complaints, with over half reporting difficulties occurring most of the time [2]. When accompanied by daytime symptoms (e.g. fatigue, cognitive impairment, mood disturbances) [5–9], sleep disturbances may meet criteria for a clinical disorder [10, 11]. In later life, sleep problems can negatively affect physical and mental health outcomes, neurological health, as well as quality of life, and mortality risk [1, 12–23]. Growing research highlights the role of the gut microbiome in health and disease, yet little work has examined its relationship to sleep health and insomnia, or conversely, the effect of sleep on the gut microbiome, representing a critical gap in understanding.

The human gastrointestinal tract contains many diverse types of microorganisms, and their combined genetic material is referred to as the gut microbiome [1]. The composition and diversity of these microbes are dynamic across the life course and susceptible to environmental as well as human genetic influence [2]. The gut microbiome may play a critical role in many facets of well-being, including neurological and behavioral health [3–5], in part through bidirectional gut-brain communication that may influence sleep and circadian rhythms [6–8].

Several emerging lines of research support a close link of the gut microbiome to sleep and circadian rhythms [9–14]. Evidence from animal research suggests that sleep disturbance alters gut microbiota composition and associated immune and metabolic markers. For example, Poroyko et al. [15] and Bowers et al. [16] exposed mice to sleep fragmentation and observed significant changes in microbiome composition and metabolites. Findings from experimental human studies have been mixed in terms of showing an association between sleep deprivation and the gut microbiome, with Benedict et al. [17] finding that two nights of partial sleep deprivation induced changes in the microbiome, while Zhang et al. [18] did not find such a link. Observational studies have reported that sleep quality is associated with microbial differences, especially in diversity [24–26]. Potential pathways linking sleep and the gut microbiome include alterations in immune function, cortisol regulation, dietary intake patterns, and microbiota-produced metabolites such as short-chain fatty acids [8, 9, 14, 17, 18, 27].

Despite these findings, key gaps remain. Prior human studies have generally included small samples, limiting statistical power and generalizability; have often relied on limited sleep assessments rather than evaluating specific insomnia-related symptoms alongside objective sleep metrics; and have provided limited species-level resolution of sleep–microbiome associations. These gaps are particularly important in older adults, who may exhibit aging-related alterations in the gut microbiome and among whom sleep disturbances are common. To address these gaps, we examined associations of self-reported sleep features with gut microbiome diversity and species-level composition among 869 older adults. Within a subset of 332 participants, we also examined associations of actigraphy-derived sleep metrics with gut microbiome diversity and composition. We hypothesized that poorer sleep health, including more frequent insomnia symptoms and daytime sleepiness as well as less favorable actigraphy-derived sleep metrics, would be associated with lower alpha diversity and differences in beta diversity and species-level microbial composition. Because self-reported sleep and actigraphy capture related but distinct aspects of sleep health, we expected partial concordance across modalities, but not complete agreement across individual measures. Given limited prior evidence at the species level, analyses of individual taxa were considered hypothesis-generating.

## Materials and methods

### Study population

We conducted a cross-sectional study using data from the Baltimore Longitudinal Study of Aging (BLSA), a cohort study administered by the Intramural Research Program of the National Institute on Aging, and the longest-running U.S. study of human aging [28]. Since its inception in 1958, the BLSA has enrolled over 3200 volunteers who are followed over time and assessed every 1–4 years, depending on their age (older individuals are assessed more frequently). During BLSA visits, participants undergo extensive evaluations of their health, function, and cognition. Between 2013 and 2019, the BLSA collected fecal specimens from 928 participants, resulting in 1841 fecal samples. We obtained microbiome sequencing data from 1810 of these samples.

We restricted our analytic sample to participant visits with complete data on self-reported sleep and key covariates (i.e. covariates included in the multivariable regression analyses). A comparison of those who were and were not included is shown in Table S1. Those who were not included in the analysis were younger and less likely to report white race. Sleep characteristics were not compared as excluded participants had high levels of missingness among self-report sleep measures. This resulted in 1567 fecal sample observations from 869 participants. For participants with more than one observation, we restricted to a single observation per participant, consisting of either the earliest available timepoint or, if a participant had actigraphy measures at any visit, we selected the earliest timepoint with actigraphy data. Sleep data and stool data were collected at the same visit, precluding any causal interpretations of associations. This resulted in cross-sectional samples of 869 participants for self-reported sleep analyses (i.e. our primary analysis) and a subset of 332 participants for actigraphy-measured sleep analyses (i.e. our secondary analysis).

### Predictors

#### Self-reported sleep measures

Self-reported sleep was assessed using a version of the Women’s Health Initiative Insomnia Rating Scale (WHIIRS) [29]. Participants were asked to report the frequency of the four following insomnia symptoms over the past month: “have trouble falling asleep (within 30 minutes),” “wake up several times at night,” “wake up earlier than you planned to,” and “have trouble getting back to sleep after you woke up too early.” Participants used a 5-point Likert scale to indicate the frequency of symptoms: (0) No, not in past 4 weeks, (1) Yes, <1/week, (2) Yes, 1-2 times/week, (3) Yes, 3-4 times/week, (4) Yes, 5+ times/week. Typical sleep quality during the past 4 weeks was also self-reported as (0) Very sound/restful, (1) Sound/restful, (2) Average quality, (3) Restless, or (4) Very restless. We combined the Restless and Very Restless categories due to small sample sizes in these reported sleep quality categories. The global WHIIRS score, ranging from 0–20, was calculated by summing the scores from these five items, with higher scores indicating greater severity of insomnia [29]. We analyzed the global WHIIRS score both as a continuous measure and as a dichotomous cut-off for insomnia (defined as having a global WHIIRS score ≥ 9) [30]. A cut-off of 9 points for insomnia has previously been used due its best balance of specificity and sensitivity [29]. Using both the dichotomous insomnia cut-off and the continuous score allows us to look at clinically meaningful scores (i.e. those exceeding the insomnia cut-off) as well as a dose–response relationship along the continuous score range. Additionally, because the several items used to generate the global WHIIRS score cover different aspects of insomnia, item-specific associations may be of interest. Participants also reported their frequency of excessive daytime sleepiness using the same 5-point Likert scale as above and reported their sleep duration (hours) in the following categories: 5 or fewer; more than 5, up to 6; more than 6, up to 7; more than 7 hours. These two latter variables are not part of the WHIIRS.

#### Actigraphic sleep parameters

Actigraphy data were collected using the Actiwatch-2 device (Philips Respironics, Bend, OR), which participants were asked to wear on their nondominant wrist for seven consecutive 24-hour periods at each study visit. Trained staff used event markers, sleep diaries, and light data to identify the time that participants were in bed with the intention of sleeping, and a validated algorithm was applied to derive sleep parameters across valid nights [31]. Four components of actigraphy-measured sleep were included in this analysis: total sleep time (modeled as continuous time and by tertiles [tertiles were used to balance the sample size across sleep categories]), wake after sleep onset (continuous), sleep onset latency (continuous), and sleep efficiency (continuous). For statistical testing, we tested the logit2 transformation of sleep efficiency; a one-unit increase in this metric can be interpreted as a doubling in the efficiency of sleep. Mathematically, this logit transformation is the $\log \left(\frac{p}{1-p}\right)=\log \left(\frac{efficiency}{1- efficiency}\right)=\log \left(\frac{efficiency}{inefficiency}\right)$, where “inefficiency” is defined as 1*-efficiency*.

### Fecal sample collection and processing

During study visits, participants were instructed on how to self-collect their stool by depositing it directly into a collection bowl placed over a toilet. Study staff transferred these samples into an empty tube and stored samples at 4°C. Samples were transferred to the clinical lab within 24 hours, where 2 mL of fecal material were aliquoted into a 50 mL Falcon tube (without buffer) and stored at –80°C until later processing.

Samples were sent to Diversigen® (Minneapolis, MN) for DNA extraction and shallow shotgun sequencing. Diversigen® performed DNA extraction from fecal samples (Qiagen PowerFecal Pro extraction kit), library preparation (Nextera library preparation kit, Illumina, CA), and sequencing of 2x150 bp paired-end reads (NextSeq, Illumina, CA). We processed raw fastq files using the bioBakery3 workflow [32] and performed read-level quality control using KneadData (v0.10.0) and Trimmomatic (v0.39) [33]. We assigned taxonomy using MetaPhlAn3 (v3.0.7). We removed viral genomic data and eukaryotic genomic data due to poor annotation and low prevalence in these samples, respectively. Further details about fecal sample collection and microbiome data can be found in earlier BLSA manuscripts [34, 35].

### Covariates

As part of the BLSA interview, participants provided health and clinical information. Participants reported their age (years), sex (male, female), and race (white, black or African-American, American Indian or Alaska Native, Asian, Native Hawaiian or other Pacific Islander, or multiracial). For race categories, we combined non-white and non-black participants into an “Other” category due to sparse data in some categories. Height and weight were used to calculate body mass index (BMI; kg/m^2^). We assessed depressive symptoms using the Center for Epidemiological Studies-Depression (CES-D), a short self-report scale that has high reliability, strong correlations with clinical ratings of depression, and has been used extensively in the epidemiologic literature [36]. For each of the 20 items, participants reported the frequency of symptoms associated with depression. Potential scores range from 0–60, with higher scores indicating greater depressive symptomology. In this analysis, we removed the sleep-related item (“my sleep was restless”) from the CES-D score to reduce direct symptom overlap between depressive symptoms and sleep measures, yielding 19 total items. We also assessed educational attainment (years), Mediterranean diet score (MDS), and physical activity (sedentary, low, medium, and high). The MDS was calculated using nine components: six beneficial food groups (vegetables, fruits and nuts, whole grains, fish, legumes, and monounsaturated to saturated fat ratio), two detrimental components (dairy, red and processed meats), and alcohol. Participants received one point for each component if their intake aligned with a Mediterranean pattern (above the sex-specific median for beneficial foods, below for detrimental ones, and moderate alcohol intake), yielding a total score from zero to nine, with higher scores indicating greater adherence [37]. We restricted diet data to individuals with non-missing diet or improbable energy intakes (for females, reporting <500 or > 3500 kcal/day; for males, reporting <700 or > 4500 kcal/day), resulting in *n* = 547 participants with diet data in self-reported sleep analyses and *n* = 198 in actigraphy measured sleep analyses.

We adjusted multivariable regression models for age, sex, physical activity, BMI, education level, and depression score; for models with richness metrics as the dependent variable, we additionally adjusted for unrarefied sequencing depth. We also assessed the influence of diet in a sensitivity analysis.

### Statistical methods

#### Sample characteristics

We performed all analyses and visualizations using R v.4.5.1 [38]. We present sample characteristics for the primary and secondary analytic samples as mean (standard deviation), median (interquartile range), or *N* (%). We stratified characteristics by tertiles of WHIIRS score (primary analytic sample) and by tertiles of actigraphy-measured total sleep time (secondary analytic sample). We tested for trends across tertiles using linear regression models for continuous variables, Cochran–Armitage trend tests for binary variables (sex), and Cochran–Mantel–Haenszel tests for multi-categorical variables (race, physical activity, and self-reported sleep measures).

#### Diversity analyses

Prior to diversity analyses, we normalized microbiome sample library sizes to a depth of 140 000 using repeated rarefaction (average of 10 rarefactions without replacement). We chose to use repeated rarefaction to 140 000 reads to standardize sampling across samples while preserving compatibility with commonly used ecological diversity metrics (e.g. Shannon diversity and UniFrac distances), which assume comparable library sizes. The selected depth of 140 000 reads in our study reflects a balance between retaining the majority of samples and ensuring sufficient sequencing depth to capture within-sample diversity, based on the inspection of read count distributions and rarefaction curves. We acknowledge that alternative normalization approaches, including centered log-ratio (CLR) transformations, are increasingly used, particularly for differential abundance testing. However, these approaches are less directly applicable to diversity metrics like UniFrac, which are sensitive to uneven sampling depth. For this reason, rarefaction remains a standard and appropriate approach for diversity analyses in microbiome studies [39]. For within-sample alpha diversity measures, we calculated two richness metrics (the number of observed species and Faith’s phylogenetic diversity), the Pielou’s evenness metric, and the Shannon diversity index. We assessed relationships between self-reported or actigraphy-measured sleep measures (independent variables) and alpha diversity metrics (dependent variables) using linear regression models; separate models were run for each sleep measure and each alpha diversity metric. Models were adjusted for age, sex, physical activity, BMI, education level, and depression score; for models with richness metrics as the dependent variable, we additionally adjusted for unrarefied sequencing depth.

For between-sample beta diversity metrics, we calculated Bray–Curtis dissimilarity, weighted UniFrac, and unweighted UniFrac. We tested associations of sleep measures with beta diversity metrics using permutational analysis of variance (PERMANOVA) testing. We performed PERMANOVA analyses using the adonis2 function from the vegan package [40], using 9999 permutations. We analyzed models separately for each beta diversity metric and sleep measure, and we adjusted models for age, sex, physical activity, BMI, education level, and depressive symptoms. For categorical sleep predictors, prior to PERMANOVA testing, we tested for significant differences in dispersion between categories; for measures where significant dispersion was identified, we note these along with our PERMANOVA findings and caution that those PERMANOVA results may be due to differences in dispersion rather than true categorical differences.

#### Differential abundance analyses

For differential abundance testing, we used the Analysis of Compositions of Microbiomes with Bias Correction 2 (ANCOM-BC2) method (v2 13.2) [41]. ANCOM-BC2 is a log-linear compositional regression framework, which attempts to mitigate sampling fraction and sequencing efficiency biases when comparing the differential abundances of taxa. To remove the effect of rare taxa, we first filtered out species with a low prevalence (i.e. species present in <10 per cent of participants) and with a low relative abundance (i.e. species with a mean relative abundance of <0.1 per cent of the study sample); we performed this filtering step separately for the primary and secondary analytic samples. We included sleep measures as predictors in the ANCOM-BC2 models, including age, sex, physical activity, BMI, education level, and depressive symptoms as covariates. We analyzed models separately for each sleep measure.

We analyzed ANCOM-BC2 models in three steps to identify: (1) species that were absent in any sleep categories (i.e. structural zeros); (2) associations of non-absent species with sleep measures; and (3) patterns of non-absent species trends over the ordered sleep categories. We illustrate our approach in Figure 1. We performed these steps because species that are determined to be structurally absent (Step 1) are not further analyzed for other types of associations (Steps 2 and 3). In Step 1, we analyzed categorical sleep variables using ANCOM-BC2 with structural zero detection, using both equations from the ANCOM-II procedure [42]. Information on species that were determined to be structurally absent in sleep categories was stored. In Step 2, we re-analyzed all categorical and continuous sleep ANCOM-BC2 models without structural zero detection. For categorical measures, we used the category for the best sleep measure as the referent group for comparisons (6–7 hours for sleep duration; Very Sound/Restful for sleep quality; no frequency of insomnia symptoms in the past month for WHIIRS items; middle tertile of actigraphy-measured sleep time). Associations were defined as significant using an FDR-corrected *q*-value <0.05 and determined as robustly statistically significantly associated if they further passed the ANCOM-BC2 pseudocount sensitivity analysis. We retained all species associations with self-reported and actigraphy-measured sleep, except for those that were determined to be structural zeros in Step 1. In Step 3, we tested for linear and U-shaped patterns across sleep categories using ANCOM-BC2’s pattern analysis methodology without structural zero detection. We determined trends as statistically significant if they passed the FDR-corrected *q*-value <0.05 and the ANCOM-BC2 pseudocount sensitivity analysis. We retained all trend associations, except for trends that included structural zeros identified in Step 1.

For both diversity and differential abundance analyses, all sleep predictors (whether self-reported or actigraphy-measured) were entered into separate models. No two sleep predictors were entered into the same model. In addition, we assessed collinearity using variance inflation factors (VIFs) and did not find evidence of concerning collinearity in our primary covariate-adjusted models (all VIFs <1.2).

#### Sensitivity and exploratory analyses

Because *Eubacterium* sp. CAG:251 emerged from the differential abundance analyses across both self-reported and actigraphy-measured sleep measures, we conducted an exploratory, post hoc targeted analysis of this selected taxon using traditional modeling approaches. We used log-binomial regressions to test associations of sleep metrics with the presence or absence of detected *Eubacterium sp.* CAG:251. If the log-binomial regressions did not initially conserve, we first fit the models using a poisson regression with log link, and used these coefficients as starting coefficients for the log-binomial models. Among participants with any detectable abundances, we further tested associations of sleep metrics with the CLR transformed abundances using linear regression models. Log-binomial and linear regression models were adjusted for age, sex, BMI, physical activity, education level, and depressive symptoms. Because this targeted analysis was data-driven and focused on a taxon selected from the initial ANCOM-BC2 results, *p*-values and confidence intervals should be interpreted cautiously.

We also conducted a sensitivity analysis, among the participants with dietary intake data (*n* = 547 for self-reported sleep; *n* = 198 for actigraphy measured sleep) in which we additionally adjusted for the alternative MDS and energy intake, and compared these results to our main findings.

Lastly, we performed a sensitivity analysis excluding two participants who each had two nights with no sleep detected by actigraphy. However, excluding them did not materially change results, so we retained them.

## Results

### Demographic and clinical characteristics

We report participant characteristics by tertile of WHIIRS global score in Table 1. Participants had a mean age of 70.7 years (SD = 13.2), 54.8 per cent were female, 66.9 per cent were white, 25.9 per cent black or African American, and 7.2 per cent identified as another race; 255 participants (29.3 per cent) had WHIIRS scores ≥9, consistent with the insomnia cut-off. Higher WHIIRS scores were associated with several clinically interpretable gradients: mean age increased from 68.4 years in the lowest WHIIRS category to 72.5 years in the highest; median CES-D score, excluding the sleep item, increased from 2.5 to 4.0; and self-reported sleep duration shifted toward shorter sleep, with the proportion reporting >7 hours decreasing from 51.6 per cent to 28.6 per cent and the proportion reporting <5 hours increasing from 1.6 per cent to 13.7 per cent. Race and physical activity also differed across WHIIRS categories, whereas sex, educational attainment, BMI, and MDS did not.

**Table 1:** Sample characteristics for participants, by tertiles of WHIIRS Score

	WHIIRS Score 0-4 (*N* = 308)	WHIIRS Score 5-8 (*N* = 306)	WHIIRS Score 9+ (*N* = 255)	Linear trend *p*-value
**Age (years)**	68.4 (14.5)	71.5 (12.6)	72.5 (12.0)	.0002
**Race [*N* (%)]**				
White	186 (60.4)	227 (74.2)	168 (65.9)	.0032
Black	100 (32.5)	57 (18.6)	68 (26.7)	
Other	22 (7.1)	22 (7.2)	19 (7.5)	
**Sex [*N* (%)]**				
Female	169 (54.9)	167 (54.6)	140 (54.9)	.9979
Male	139 (45.1)	139 (45.4)	115 (45.1)	
**Educational attainment category**				
Post college	200 (64.9%)	193 (63.1%)	161 (63.1%)	.5112
Some college or college grad	99 (32.1%)	101 (33.0%)	79 (31.0%)	
High school or less	9 (2.9%)	12 (3.9%)	15 (5.9%)	
**BMI (kg/m** ^**2**^ **)**	27.5 (4.87)	27.0 (4.57)	27.6 (5.17)	.8890
**MDS**	4.42 (1.62)	4.47 (1.56)	4.26 (1.72)	.4085
Missing [N (%)]	112 (36.4%)	108 (35.3%)	102 (40.0%)	
**Energy intake (kcal/day)**	1800 (729)	1940 (720)	1940 (698)	.0566
Missing	112 (36.4%)	108 (35.3%)	102 (40.0%)	
**Physical activity [*N* (%)]**				
Sedentary	30 (9.7)	26 (8.5)	30 (11.8)	.0434
Low	113 (36.7)	103 (33.7)	113 (44.3)	
Medium	92 (29.9)	106 (34.6)	58 (22.7)	
High	73 (23.7)	71 (23.2)	54 (21.2)	
**CES-D score (without sleep item)**	2.50 [1.00, 5.00]	3.00 [1.00, 5.00]	4.00 [2.00, 8.00]	<0.0001
**Sleep duration [*N* (%)]**				
>7 hours	159 (51.6)	158 (51.6)	73 (28.6)	<.0001
6–7 hours	106 (34.4)	112 (36.6)	84 (32.9)	
5–6 hours	38 (12.3)	33 (10.8)	63 (24.7)	
<5 hours	5 (1.6)	3 (1.0)	35 (13.7)	
**Excessive daytime sleepiness [*N* (%)]**				
never	265 (86.0)	216 (70.6)	157 (61.6)	<.0001
<1/week	16 (5.2)	35 (11.4)	28 (11.0)	
1–2/week	13 (4.2)	28 (9.2)	39 (15.3)	
3–4/week	4 (1.3)	11 (3.6)	20 (7.8)	
5+/week	10 (3.2)	16 (5.2)	11 (4.3)	
**Trouble falling asleep within 30 minutes [*N* (%)]**				
no	236 (76.6)	131 (42.8)	64 (25.1)	<.0001
<1/week	51 (16.6)	73 (23.9)	32 (12.5)	
1–2/week	17 (5.5)	77 (25.2)	93 (36.5)	
3–4/week	3 (1.0)	15 (4.9)	35 (13.7)	
5+/week	1 (0.3)	10 (3.3)	31 (12.2)	
**Wake up several times at night [*N* (%)]**				
no	191 (62.0)	36 (11.8)	7 (2.7)	<.0001
<1/week	51 (16.6)	33 (10.8)	8 (3.1)	
1–2/week	38 (12.3)	70 (22.9)	39 (15.3)	
3–4/week	11 (3.6)	40 (13.1)	52 (20.4)	
5+/week	17 (5.5)	127 (41.5)	149 (58.4)	
**Wake up earlier than planned [*N* (%)]**				
no	252 (81.8)	146 (47.7)	16 (6.3)	<.0001
<1/week	38 (12.3)	65 (21.2)	18 (7.1)	
1–2/week	15 (4.9)	73 (23.9)	77 (30.2)	
3–4/week	3 (1.0)	15 (4.9)	61 (23.9)	
5+/week	0 (0)	7 (2.3)	83 (32.5)	
**Trouble falling back asleep after waking up too early [*N* (%)]**				
no	293 (95.1)	222 (72.5)	54 (21.2)	<.0001
<1/week	13 (4.2)	52 (17.0)	28 (11.0)	
1–2/week	2 (0.6)	31 (10.1)	95 (37.3)	
3–4/week	0 (0)	1 (0.3)	45 (17.6)	
5+/week	0 (0)	0 (0)	33 (12.9)	
**Quality of typical night’s sleep [*N* (%)]**				
Very sound/restful	140 (45.5)	29 (9.5)	9 (3.5)	<.0001
Sound/restful	111 (36.0)	135 (44.1)	44 (17.3)	
Average quality	54 (17.5)	129 (42.2)	137 (53.7)	
Restless/very restless	3 (1.0)	13 (4.2)	65 (25.5)	

*Note:*  Table 1 displays sample characteristics for BLSA participants who had both self-reported sleep measures and fecal microbiome data (*N* = 869). Data are presented as mean (standard deviation), median (interquartile range), or *N* (%), and are prevented stratified by tertiles of WHIIRS global score. Linear trend *p*-value across tertiles of WHIIRS global score determined using linear regression (continuous variables), Cochran-Armitage trend test (sex), and Cochran–Mantel–Haenszel test (race, physical activity, and self-reported sleep measures. Abbreviations: WHIIRS = Women’s Health Initiative Insomnia Rating Scale; BLSA = Baltimore Longitudinal Study of Aging; BMI = body mass index; CES-D = Center for Epidemiological Studies Depression scale.

### Associations of self-reported sleep with diversity measures

In Table 2, we report unadjusted and adjusted associations of self-reported sleep measures with Shannon diversity index, our primary measure of microbiome alpha diversity; we report associations with alternative measures of alpha diversity, including microbial richness (observed species, Faith’s phylogenetic diversity) and Pielou’s evenness in Tables S2–S4. We observed an association between the worst versus best category of “trouble falling asleep” with a higher Shannon index (Table 2) and Pielou’s evenness (Table S4). These associations suggested (albeit not statistically significantly) a dose–response relationship between increasing difficulty in falling asleep with higher microbiome Shannon index (*β*: 0.06, 95% CI: –0.00–0.11) and evenness (*β*: 0.05, 95% CI: –0.00–0.11). Categories of “trouble falling asleep” were not associated with richness metrics (observed species and Faith’s phylogenetic diversity), though estimates were in a similar direction as with Shannon index and Pielou’s evenness. While other associations were noted (Table 2 and Tables S2–S4), these were not suggestive of a dose–response relationship.

**Table 2:** Difference in microbiome Shannon diversity according to self-reported measures of sleep

Sleep predictor	Model	>7 hours vs. 6–7 hours	5–6 hours vs. 6–7 hours	<5 hours vs. 6–7 hours		Continuous
Sleep duration	M1	**0.15 (0.00–0.30)**	0.16 (-0.04–0.36)	-0.07 (-0.39–0.25)		0.04 (-0.04–0.12)
	M2	0.15 (-0.00–0.30)	0.18 (-0.02–0.39)	-0.03 (-0.36–0.30)		0.05 (-0.03–0.13)
Sleep predictor	Model	Sound/restful vs. very sound/restful	Average vs. very sound/restful	Restless/very restless vs. very sound/restful		Continuous
Sleep quality	M1	-0.02 (-0.20–0.17)	0.03 (-0.16–0.21)	0.11 (-0.15–0.38)		0.03 (-0.04–0.10)
	M2	-0.01 (-0.20–0.18)	0.03 (-0.15–0.22)	0.15 (-0.12–0.42)		0.04 (-0.04–0.11)
Sleep predictor	Model	Insomnia (Yes vs. No)	—	—		Continuous WHIIRS Score
WHIIRS insomnia/global score	M1	-0.01 (-0.16–0.13)	—	—		0.01 (-0.01–0.02)
	M2	0.00 (-0.15–0.15)	—	—		0.01 (-0.01–0.03)
Sleep predictor	Model	<1/week vs. Not in past month	1-2 times/week vs. not in past month	3-4 times/week vs. not in past month	5+ times/week vs. Not in past month	Continuous
Trouble falling asleep	M1	0.01 (-0.18–0.19)	-0.01 (-0.18–0.16)	0.13 (-0.15–0.42)	**0.38 (0.07**–**0.70)**	0.04 (-0.04–0.12)
	M2	0.01 (-0.18–0.19)	-0.01 (-0.18–0.17)	0.15 (-0.14–0.43)	**0.41 (0.09**–**0.73)**	0.06 (-0.00–0.11)
Wake several times/night	M1	0.02 (-0.22–0.26)	-0.01 (-0.22–0.19)	-0.20 (-0.43–0.03)	0.09 (-0.08–0.26)	0.01 (-0.03–0.05)
	M2	0.00 (-0.24–0.25)	-0.04 (-0.25–0.17)	-0.21 (-0.45–0.02)	0.08 (-0.10–0.26)	0.01 (-0.03–0.05)
Wake earlier than planned	M1	0.16 (-0.04–0.37)	**0.19 (0.01**–**0.37)**	-0.12 (-0.37–0.12)	0.03 (-0.19–0.26)	0.01 (-0.04–0.05)
	M2	0.16 (-0.04–0.37)	**0.20 (0.02**–**0.38)**	-0.11 (-0.35–0.13)	0.07 (-0.16–0.30)	0.01 (-0.03–0.06)
Trouble falling asleep after waking	M1	0.07 (-0.15–0.29)	-0.06 (-0.25–0.14)	-0.02 (-0.32–0.29)	0.11 (-0.24–0.46)	0.04 (-0.04–0.12)
	M2	0.07 (-0.15–0.29)	-0.04 (-0.24–0.15)	0.01 (-0.30–0.31)	0.14 (-0.22–0.50)	0.01 (-0.05–0.07)
Excessive sleepiness	M1	0.16 (-0.08–0.39)	0.02 (-0.21–0.25)	-0.22 (-0.56–0.12)	0.10 (-0.23–0.43)	0.00 (-0.06–0.06)
	M2	0.15 (-0.08–0.39)	0.01 (-0.22–0.25)	-0.23 (-0.58–0.12)	0.14 (-0.20–0.48)	0.00 (-0.06–0.07)

*Note:*  Table 2 displays unadjusted and adjusted associations of self-reported sleep measures with the alpha diversity measure Shannon Diversity. Associations are reported as the *β* (95% confidence interval) from linear regression models with sleep measures as the independent variable and normalized Shannon diversity index as the dependent variable. Self-reported sleep measures were analyzed both categorically (comparing progressively worsening sleep categories [1→4] to the best sleep category [0]) and as a continuous measure. M1: Unadjusted associations. M2: M1 + age, sex, physical activity, BMI, education level, and CESD depression score. Bolded estimates are statistically significant (*p* < 0.05). Abbreviations: WHIIRS = Women’s Health Initiative Insomnia Rating Scale; BMI = body mass index.

We report associations of self-reported sleep measures with measures of microbiome beta diversity in Table S5. After adjustment for covariates, we found associations for the frequencies of Trouble Falling Asleep and Excessive Sleepiness with Bray–Curtis dissimilarity; and Trouble Falling Asleep After Waking and Excessive Sleepiness with unweighted UniFrac (though associations with Trouble Falling Asleep After Waking could be due to heterogeneous dispersions between frequencies-per-week groupings). Notably for Excessive Sleepiness symptoms, it was primarily the groups reporting moderate symptoms (1–2 times per week or 3–4 times per week) who had significantly different compositions compared to those never reporting excessive sleepiness. *R*^2^ values, indicating the variance explained, are included in Table S5; these explained 0.1 per cent of the variance.

### Association of self-reported sleep measures with differentially abundant species

We estimated associations of categories of self-reported sleep measures with microbial species using ANCOM-BC2 and present results for each model in Tables S6–S8. In Figure 1, we display the 16 taxa significantly associated with at least one self-reported sleep measure (FDR *q* < 0.05) or had a significant trend across worsening sleep symptom categories. While no taxon was universally associated with worse sleep symptoms, some interesting patterns emerged. Notably, *Eubacterium* sp. CAG:251 and *Acidaminococcus intestini* were more likely to be low abundant or undetected in participants who reported the higher frequencies of Trouble Falling Asleep (*Eubacterium* sp. CAG:251 only*)*, Trouble Falling Asleep After Waking, and Excessive Sleepiness. In trend tests (Table S8–S9), more frequent Trouble Falling Asleep After Waking was associated with greater abundance of *Erysipelatoclostridium ramosum,* and there were umbrella-shaped trend between frequencies of Trouble Falling Asleep with *Eubacterium* sp. CAG:180; Trouble Fall Asleep After Waking with *Escherichia coli*; Wake Several Times per Night with *Firmicutes bacterium* CAG:424; and Excessive Sleepiness with *Bifidobacterium adoloscentis*.

**Figure 1:**
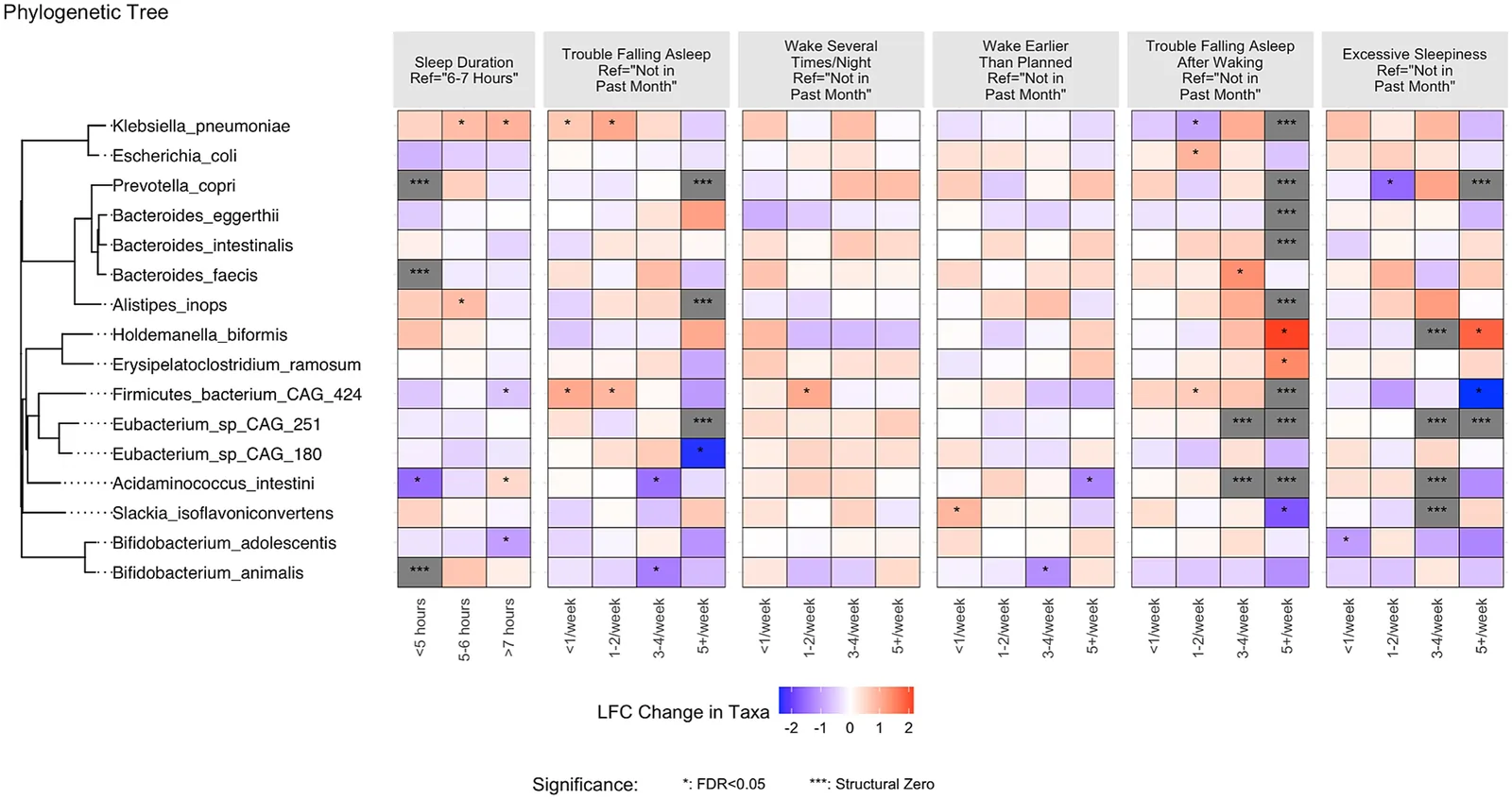
**Heatmap displaying associations of self-reported sleep measures with microbial differential abundance.** Displayed taxa are those which were absent (i.e. a structural zero) in one or more sleep categories (denoted with ^***^), or which had a statistically significant trend across sleep categories (detailed in Table S5; FDR < 0.05 and passed pseudocount sensitivity test). Displayed self-reported sleep categories include those in which at least one significant taxonomic association (structural zero or trend) was detected. Also plotted are suggestive associations (denoted with^*^), which are associations that passed the FDR <0.05 threshold but did not pass the robust pseudocount sensitivity test. Models were adjusted for age, sex, physical activity, education level, BMI, and depression. Abbreviations: WHIIRS = Women’s Health Initiative Insomnia Rating Scale; BMI = body mass index; LFC = logfold change; ANCOMBC2 = Analysis of Compositions of Microbiomes with Bias Correction 2, FDR = false discovery rate.

### Actigraphy measured sleep

We next investigated whether objective actigraphy-based sleep metrics were associated with the microbiome. Sample characteristics by tertiles of total sleep time for the actigraphy subsample are shown in Table S10, and a comparison of this subsample to the larger sample is shown in Table S11. The actigraphy subsample had more white and fewer black individuals than the larger sample, and a lower CESD score (interquartile range was wider in the self-report sample). Actigraphy was available for 332 of 869 participants. Compared with participants without actigraphy, the actigraphy subsample included a higher proportion of white participants (72.0 per cent vs. 63.7 per cent) and a lower proportion of black participants (22.0 per cent vs. 28.3 per cent; *p* = .041). The CES-D distribution also differed modestly, with the same median but a lower upper quartile in the actigraphy subsample (median [IQR]: 3.0 [1.0, 6.0] vs. 3.0 [1.0, 7.0]; *p* = .0155). WHIIRS scores were the same across groups (median [IQR]: 6.0 [3.0, 9.0] in both groups; *p* = .206), and other measured characteristics were broadly similar. We found no significant associations of any of the actigraphy-measured sleep variables with alpha or beta diversity indices. This included a null association between total sleep time, categorized into tertiles, and all diversity indices (Tables S12 and S13). We estimated associations of actigraph-measured sleep with relative abundance of microbial species using ANCOM-BC2 (Figure 2; all results included in Tables S14–S16). We plot associations for all taxa which were significant in the self-reported sleep analysis, regardless of their significance in the actigraphy analysis. No taxon was significant after both FDR correction and pseudocount sensitivity testing, however, several taxa were significant at the FDR *q* < 0.05 threshold. Of note, actigraphy-measured sleep was significantly associated with *Eubacterium* sp. CAG:251; specifically, amount of time spent awake after sleep onset was inversely associated with abundance of *Eubacterium sp.* CAG:251, and sleep efficiency was positively associated with abundances.

**Figure 2:**
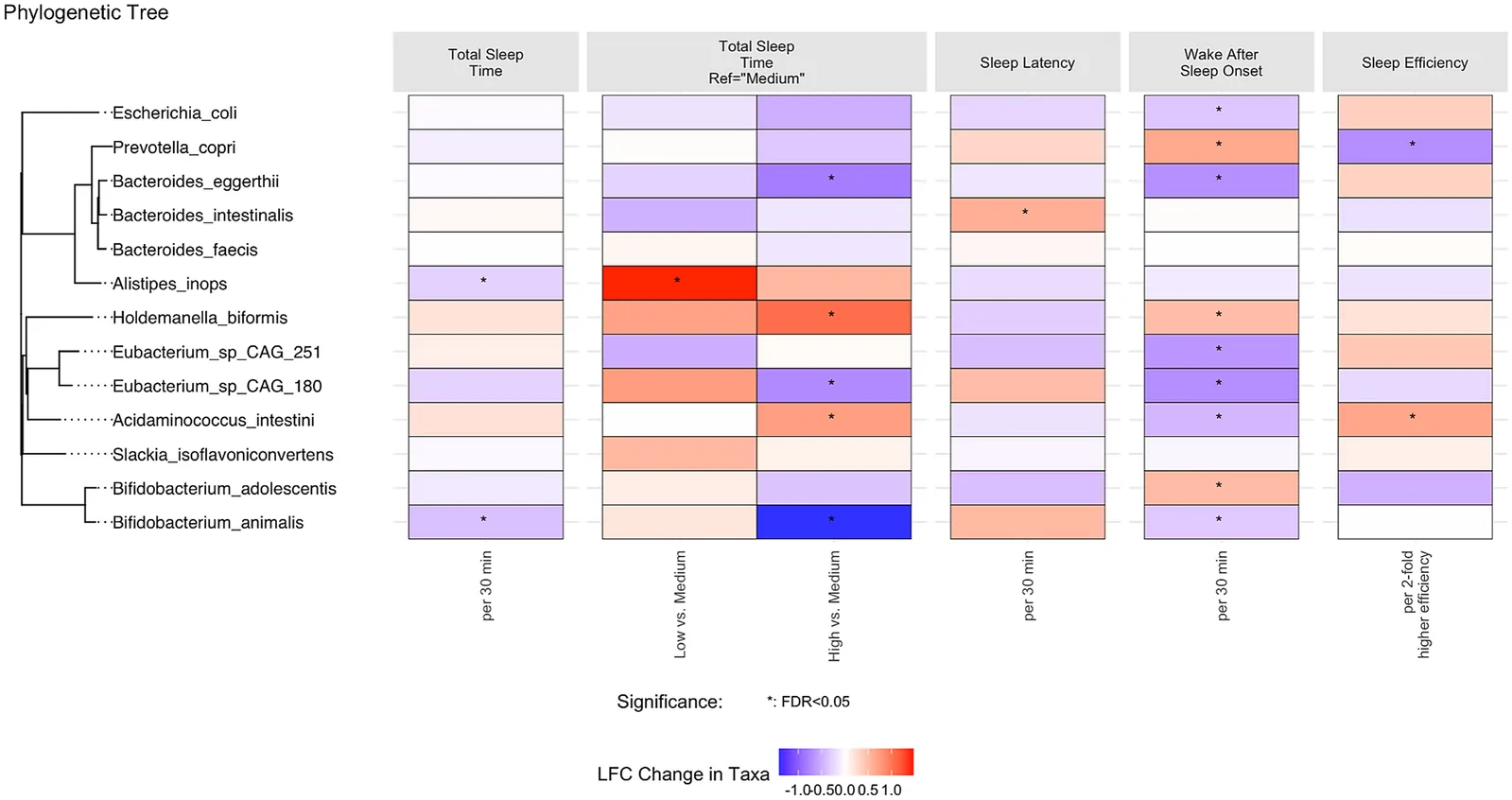
**Heatmap displaying associations of actigraphy sleep measures with microbial differential abundance.** Displayed taxa include those which were structurally absent or exhibited a trend in self-reported sleep measures (included for comparison, with the exception of *Klebsiella pneumoniae*, *Firmicutes bacterium CAG:424*, and *Erysipelatoclostridium ramosum*, which were not present in this subsample after filtering thresholds were applied). Also plotted are suggestive associations (denoted with^*^), which are associations that passed the FDR <0.05 threshold but did not pass the robust pseudocount sensitivity test. Models were adjusted for age, sex, physical activity, BMI, education level, and depression. Abbreviations: BMI = body mass index, LFC = logfold change, ANCOMBC2 = Analysis of Compositions of Microbiomes with Bias Correction 2, FDR = false discovery rate.

#### Sensitivity and exploratory analyses

Given that multiple self-reported sleep metrics were associated with *Eubacterium* sp. CAG:251, we further investigated, in exploratory post hoc analyses, the association of actigraphy-measured sleep with the prevalence and abundance of *Eubacterium* sp. CAG:251 (Figure 3 and Table S17). Each doubling of the sleep efficiency ratio is associated with a 2.15-fold higher prevalence of *Eubacterium* sp. CAG:251 (i.e. doubling sleep efficiency is associated with roughly doubling the prevalence of this *Eubacterium* species). Each 30-minute higher WASO is associated with a 0.5-fold prevalence of having *Eubacterium* sp. CAG:251, i.e. a halving of the prevalence. In line with the ANCOMBC2 results, among those with any presence of *Eubacterium* sp. CAG:251, time awake after sleep onset was inversely and significantly associated with the abundance of *Eubacterium* sp. CAG:251, while sleep efficiency was positively (albeit not statistically significantly) associated with *Eubacterium* sp. CAG:251 abundance. Because these targeted analyses focused on a taxon selected from the initial differential abundance results, *p*-values and confidence intervals should be interpreted cautiously.

**Figure 3:**
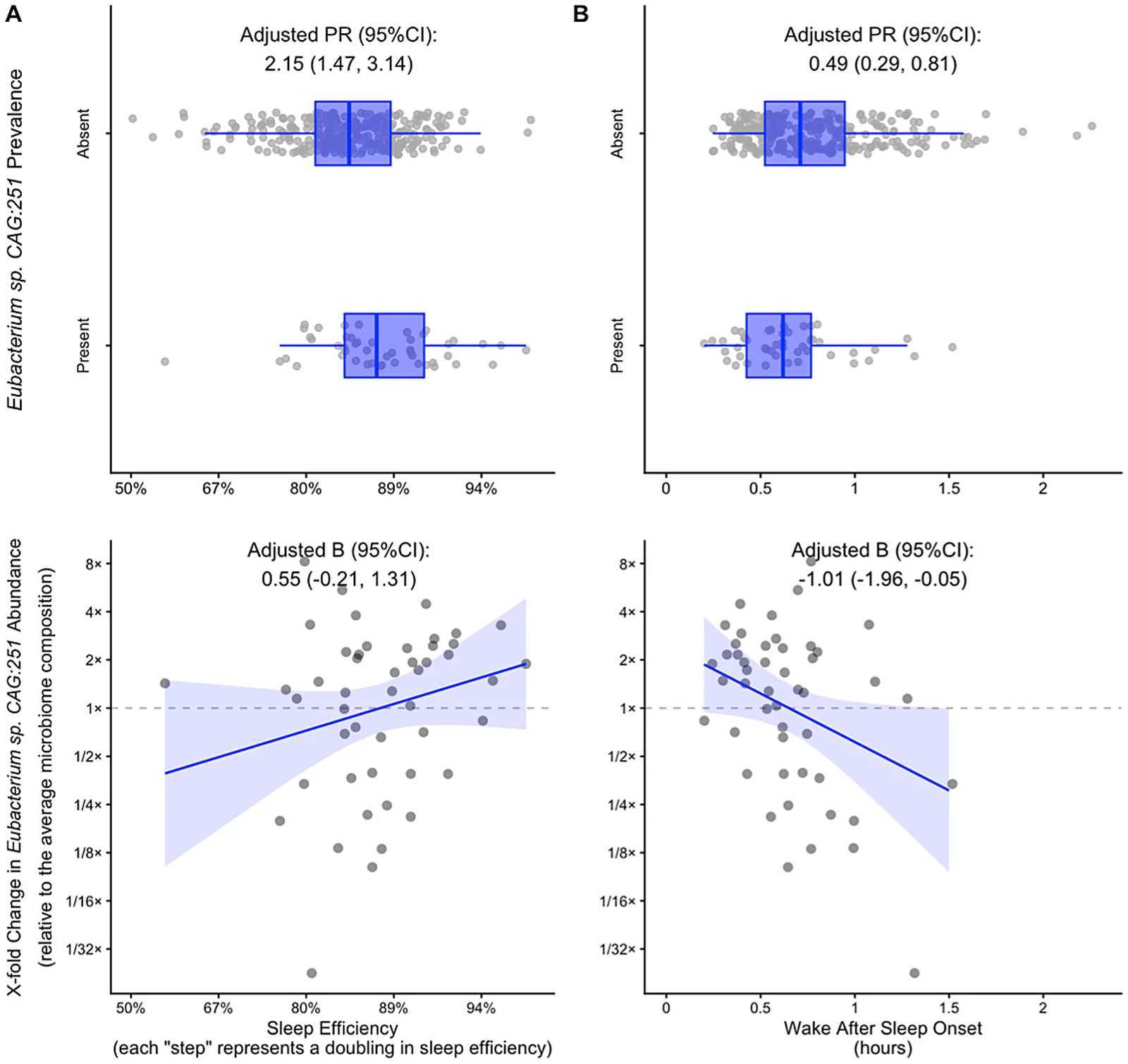
**Associations of actigraphy sleep measures with prevalence and abundance of *Eubacterium sp. CAG:251*.** Presented sleep measures include (A) sleep efficiency and (B) wake after sleep onset. The top row of each panel shows boxplots for associations of sleep metrics by prevalence of *Eubacterium sp. CAG:251*; adjusted prevalence ratios and 95% confidence intervals from log-binomial models are displayed. To provide perspective, *Eubacterium sp. CAG:251* was present in *n* = 48 (14.5%) of participants with actigraphy sleep measures. The bottom row of each panel shows associations of sleep metrics (among the *n* = 48 individuals with present *Eubacterium sp. CAG:251*) with the CLR transformed abundances, where more positive values reflect higher abundances of *Eubacterium sp. CAG:251* relative to the average microbiome composition. CLR abundances are presented on the *y*-axis as X-fold difference (e.g. “2×” is a 2-fold higher *Eubacterium sp. CAG:251* abundance relative to the average microbiome composition). Lines represent the regression lines and slopes from covariate-adjusted linear regression models and are plotted over the raw data. Log-binomial models and linear regression models were adjusted for age, sex, BMI, physical activity, education level, and depression. Abbreviations: CLR = center log ratio; PR = prevalence ratio; B = beta coefficient; CI = confidence interval; BMI = body mass index.

As a sensitivity analysis, among the subset of participants with dietary intake data available, we compared the impact of diet adjustment (using the alternative MDS and energy intake as covariates) versus no diet adjustment. The findings from these analyses were similar to those of our main analyses (Tables S18–S20).

## Discussion

In this well-characterized cohort of older adults, we found inconsistent associations between self-reported sleep disturbances and gut microbiome diversity, and no associations of objectively measured (actigraphy-based) sleep with microbiome diversity in a subset of participants. However, across both subjective and objective sleep measures, *Eubacterium* sp. CAG:251 showed a more coherent pattern: its presence and abundance were lower among individuals with more frequent self-reported wake after sleep onset and higher among those with better actigraphic sleep efficiency. Given the cross-sectional design and the number of sleep and microbiome features evaluated, these findings should be interpreted as associative and hypothesis-generating. Rather than establishing directionality, they identify *Eubacterium* sp. CAG:251 as a candidate taxon for future replication, longitudinal analyses, and mechanistic studies.

Certain *Eubacterium* species are known producers of short-chain fatty acids (SCFAs), particularly butyrate, which has been implicated in host circadian rhythms [43, 44] However, the genus *Eubacterium* consists of many diverse species, and functional evidence at the genus level or for other *Eubacterium* species should not be assumed to apply directly to *Eubacterium* sp. CAG:251, which remains poorly characterized. In an experimental study of an exercise intervention, *Eubacterium hallii* was a microbe marker in the sleep-disordered population (Pittsburgh Sleep Quality Index ≥ 7) before and following the exercise intervention, though no marker was found in the healthy population. Accordingly, the increase in this microbe was associated with healthier sleep in this population of university students [45]. Additionally, in animal models, experimentally induced sleep deprivation decreased the *Eubacterium* genus [46]. Together, these studies provide context for our observation that *Eubacterium*-related taxa may be associated with sleep health; however, our cross-sectional findings do not establish whether sleep disturbance contributes to differences in *Eubacterium* sp. CAG:251, whether this taxon influences sleep, or whether both are shaped by shared behavioral, physiological, or environmental factors. Notably, *Eubacterium* also plays a role in energy homeostasis, colonic motility, immunomodulation, and contribution to bile acid and cholesterol transformations [44]. However, because these functions have not been established specifically for *Eubacterium* sp. CAG:251 and we did not measure SCFAs, microbial gene pathways, or metabolomic profiles, these mechanisms should be interpreted as biological context rather than direct evidence of CAG:251 function.

Unlike recent actigraphic research in healthy, older Chinese adults [47], actigraphy-derived metrics such as total sleep time, sleep efficiency, and wake after sleep onset were not associated with microbial diversity or composition in our study, with the exception of *Eubacterium.* This may reflect several possibilities: (1) perceived sleep symptoms (which may capture other constructs such as anxiety or fatigue) may more closely align with gut microbial features than physiologically measured sleep; (2) actigraphy, while objective, captures limited nuance about sleep architecture [48]; (3) the gut microbiome may differentially associate with the cognitive-emotional consequences of poor sleep (e.g. fatigue, distress) more than with raw sleep parameters; and (4) we are under powered given the sample size of the actigraphy sample is smaller (though we note both the point estimates and confidence intervals were null, so the smaller sample size may not completely explain this discrepancy).

Several studies provide converging evidence that sleep quality is related to microbial differences. Anderson et al. [24] observed that in a cross-sectional survey of 37 healthy older adults without dementia or neurological disease, self-reported poorer sleep quality was associated with lower relative abundance of the phyla *Verrucomicrobia* and *Lentisphaerae*. Similarly, Smith et al. [25] found that among 26 men, higher sleep efficiency and total sleep time were positively associated with total microbial diversity and wake after sleep onset was negatively associated with microbial diversity. Further, there was a positive association between total microbial diversity and IL-6. Within-phyla richness of *Bacteroidetes* and *Firmicutes* were positively associated with sleep efficiency, IL-6, and abstract reasoning, as measured by an Abstract Matching test. Several individual taxa (*Lachnospiraceae*, *Corynebacterium*, and *Blautia*) were also associated with sleep parameters. Grosicki and colleagues [26] found that in 28 young and healthy adults, higher self-reported sleep quality was associated with greater microbial diversity and a higher *Firmicutes*:*Bacteroidetes* ratio, driven by greater relative abundance of *Blautia* and *Ruminococcus* and lower abundance of *Prevotella*.

Our findings similarly show associations between Trouble Falling Asleep and Excessive Sleepiness with beta diversity, and having trouble falling asleep with alpha diversity. Smith et al. [25] and Grosicki et al. [26] found that self-reported higher sleep efficiency, greater total sleep time, or higher self-reported sleep quality was associated with greater microbial diversity. However, we found an association in the opposite direction, such that frequent trouble falling asleep was associated with higher alpha diversity. Because our sample consisted primarily of older adults, this discrepancy may reflect life-course differences in sleep–microbiome relationships, although this interpretation is speculative. Higher diversity may not always indicate optimal gut microbial health in later life; age-related shifts could reflect resilience, remodeling, instability, or dysbiosis. Future longitudinal studies with repeated sleep and stool assessments, together with functional metagenomic or metabolomic readouts, are needed to clarify whether these diversity patterns reflect beneficial or adverse microbial changes in the aging gut.

Our study adds to emerging literature demonstrating potential links between sleep and the gut microbiome [9–14]. In terms of animal studies, Poroyko et al. showed in mice that four weeks of sleep fragmentation produced significant increases in the highly fermentative members of family *Lachnospiraceae* and *Ruminococcaceae,* and a decrease in *Lactobacillaceae* [15]. More recently, Bowers et al. [16] exposed mice to five days of sleep disruption and four days of subsequent recovery sleep and measured the fecal microbiome and metabolites over time. On the second day of recovery sleep, the sleep-disrupted mice, relative to control mice, had an increase in the *Firmicutes*:*Bacteroidetes* ratio, and decreases in the *Lactobacillus* genus, *Actinobacteria* phylum, and *Bifidobacterium* genus. Multiple microbial metabolites were also differentially abundant between the experimental and control mice [16]. Human research also supports the possibility of a link between poor sleep or sleep deprivation on the gut microbiome, though these studies have relied on small samples. Benedict et al. [18] demonstrated that after two nights of experimental partial sleep deprivation, young healthy adult males (*n* = 9) showed a higher *Firmicutes:Bacteroidetes* ratio and alterations in the abundance of families *Erysipelotrichaceae*, *Coriobacteriaceae*, and *Tenericutes*. Contrary to this study, however, Zhang et al. did not find a significant link between sleep restriction over several days and the composition of the gut microbiome in a sample of 11 adult females [17].

Our study has several notable strengths, including the integration of self-report instruments with wrist actigraphy to capture both perceived and objectively inferred sleep patterns, providing a nuanced understanding of sleep-microbiome relationships. We leveraged whole genome sequencing and analyzed a large sample relative to prior studies, adjusting for multiple potential confounders. Importantly, our focus on older adults—an underrepresented group in sleep and gut microbiome research despite a high prevalence of sleep disturbances and age-related microbiota changes—offers a rare opportunity to examine these associations in a demographically diverse, well-phenotyped population, the BLSA. Additionally, although actigraphy data were only available for a subset, our study remains, to our knowledge, the largest to date investigating microbiome links with objectively assessed sleep.

Several limitations should be noted. First, our analysis was cross-sectional, with sleep and stool data collected at the same visit; therefore, we cannot determine whether sleep disturbances preceded microbiome differences, microbiome differences influenced sleep, or both were shaped by shared factors. Although models adjusted for key covariates, residual confounding remains possible, including from diet not fully captured by the MDS, medications such as antibiotics or proton pump inhibitors, comorbidities, and unmeasured behavioral or psychosocial factors. Depressive symptoms are also particularly complex in the context of this study because they are closely linked with both sleep disturbance and the gut microbiome. Depression may confound sleep–microbiome associations, lie on the causal pathway between sleep disturbance and microbial differences, or reflect unmeasured psychosocial stressors that influence both sleep and the gut microbiome. We adjusted for depressive symptoms using a CES-D score with the sleep-related item removed to reduce direct overlap with sleep measures. While this adjustment may reduce confounding by depressive symptoms, it could also attenuate total associations if depressive symptoms are part of the pathway of interest. Given the cross-sectional design, we interpret our findings as associations rather than estimates of causal effects. Second, generalizability may be limited by selection into the BLSA, a healthy volunteer cohort, and by our restriction to participants with complete self-reported sleep and covariate data. Excluded participants were younger and less likely to report white race, and their sleep characteristics could not be compared because of substantial missingness in self-reported sleep measures. Because actigraphy data were available only for a subset of participants, actigraphy-based results may be less generalizable than those involving the full analytic sample. The actigraphy subsample was somewhat more likely to include white participants and had a slightly lower depressive symptom distribution than participants without actigraphy, raising the possibility of selection bias if actigraphy participation was related to unmeasured health, behavioral, or psychosocial factors associated with both sleep and the gut microbiome. In addition, in contrast to polysomnography, actigraphy provides objective estimates of sleep timing and continuity but is a relatively crude proxy for sleep physiology and does not capture sleep architecture, arousals, or sleep-disordered breathing. Third, although shotgun metagenomic sequencing enabled species-level analyses, sequencing depth was insufficient for accurate annotation and quantification of fungal or viral communities, which may also be relevant to sleep and health. Finally, we evaluated many sleep exposures and microbiome outcomes. While ANCOM-BC2 models used FDR correction for taxon-level analyses, we did not apply a global correction across all sleep measures, diversity metrics, taxa, and exploratory follow-up models. Therefore, isolated diversity findings and taxa-level associations, including *Eubacterium* sp. CAG:251, should be interpreted as exploratory and hypothesis-generating pending replication.

Longitudinal studies are necessary to clarify the directionality and potential mediating pathways linking sleep, the gut microbiome, depressive symptoms, and downstream health outcomes. Poor sleep or insomnia may contribute to microbial dysbiosis, which may in turn influence outcomes such as cognitive health; alternatively, gut microbial alterations may influence sleep health, depressive symptoms, or other physical and mental health outcomes. Depression is particularly important to evaluate in this framework, as it may act as a confounder, mediator, or marker of shared psychosocial stressors. Future work should leverage repeated sleep and stool assessments, including within-person analyses of temporal changes in sleep and microbiome features, to determine whether sleep changes precede microbial shifts or vice versa. In the BLSA, existing longitudinal sleep, stool, cognitive, and health data provide an opportunity to examine temporal ordering and to conduct mediation analyses for outcomes such as cognitive decline. Integration with functional metagenomic and metabolomic data, including SCFAs and other microbial metabolites, would further help test candidate biological pathways connecting sleep, the gut microbiome, and health outcomes.

## Supplementary Material

Supplementary_material_zsag217

## Data Availability

Data can be requested through the BLSA website: https://www.blsa.nih.gov/blsa-data-use.
